# Effects of Yin and Yang supplement on reproductive performance, antioxidant and immunity of dairy goats

**DOI:** 10.1080/10495398.2025.2450349

**Published:** 2025-01-19

**Authors:** Kang Wang, Zhi Yang, Fumei Yang, Guanzong Li, Yulin Sun, Gang Duan, Jun He, Wang Sun, Ke Zhou, Zhihao Xiong, Feiyan Dai

**Affiliations:** a College of Veterinary Medicine, Yunnan Agricultural University, Kunming, China; b Animal Disease Prevention and Control Center of Chuxiong, Chuxiong, China; c Rural Revitalization Service Center of Mengzhe Town, Menghai County, Xishuangbanna, China; d College of Veterinary Medicine, South China Agricultural University, Guangzhou, China; e Veterinarian, Kunming Technical Contract Accreditation and Registration Station, Kunming, China

**Keywords:** Compound Chinese medicine preparations, dairy goats, reproductive performance, regulation

## Abstract

This study aims to explore the effects of Yin and Yang Double Supplement Compound Chinese Medicine Preparations (YYSBFF) on the reproductive performance, antioxidant levels, and immunity of dairy goats. For the experiment, 36 Alps milk goats were selected and randomly divided into an experimental group and a control group, with 18 goats in each group. The ewes in the experimental group were fed with YYSBFF for 14 d prior to breeding and farrowing. The results of the experiment showed that the estrus rate, embryo acceptance rate, and lamb birth weight in the experimental group were higher than those in the control group, and the weak lamb rate was significantly lower. Moreover, the experimental group exhibited higher levels of reproductive hormones (FSH, LH), antioxidant factors (T-SOD, GSH-Px, MDA), and immunoglobulins (IgA, IgM, IgG) compared to the control group. There were no significant differences in liver and kidney function indicators (ALT, AST, TP, ALB, CREA, UREA) between the experimental and control groups (*p* > .05). These findings indicate that YYSBFF can enhance the reproductive performance of dairy goats by regulating the level of sex hormones, while also improving the body’s antioxidant and immune abilities.

## Introduction

Dairy goat farming is a low-investment, relatively stable-income industry and is an important component of the global dairy sector. Since the 1990s, the global dairy goat population has experienced rapid and continuous growth.^1^ According to the Food and Agriculture Organization of the United Nations (FAO), as of 2021, there were approximately 219 million dairy goats worldwide, making them a significant component of the dairy industry.^2^ Goats are seasonal breeders, and the duration and timing of their estrus season are influenced by various factors, including breed, feeding environment, nutrient levels, and reproductive system.^3^ The extended postpartum estrus period is the primary factor limiting the reproductive efficiency of dairy goats. Advancements in reproductive technology, such as synchronized estrus,^4^ superovulation,^5^ artificial insemination,^6^ and embryo transfer,^7^ have been developed and applied in dairy goat farming. The most commonly used superovulation regimens are ecG + CIDR or FSH+CIDR.^8^ However, it has been observed that excessive use of ecG or FSH in repeated superovulation can lead to adverse reproductive reactions, reduced egg quality, and decreased fertility. This may even result in immune system damage.^9^ The widespread implementation of MOET (Multiple Ovulation and Embryo Transfer) in goats has been hindered by the high cost of hormones and the unpredictable response of recipient animals to superovulatory hormones.^3^

The application of Chinese herbal feed additives in the field of animal husbandry has gained increasing attention.^10^^,^^11^ Compound preparations, due to their synergistic effects and multiple biological functions, offer greater potential and advantages compared to single herbal preparations.^12^^,^^13^ The regulatory role of reproductive hormones in animal reproduction, development, and growth is crucial. In the reproductive process of female animals, sex hormones directly impact estrus, ovulation, pregnancy, embryonic development, parturition, and lactation.^14^ Certain herbal additives can promote follicle development and regulate the secretion of reproductive hormones, thereby inducing estrus in female animals.^15^^,^^16^ Xia et al.^17^ used Chinese herbal medicine (CHM) to treat rat models of ovarian dysfunction with impaired chemotherapy drugs, and conventional gonadotropin-releasing hormone agonist (GnRHa) therapy was used as a control. The results showed that body weight, fertility, days in heat, hormone levels, and ovarian weight were all restored with the administration of CHM in these rat models.

The health status of animals is closely related to the body’s antioxidant function and immunity. Normally, the body’s antioxidant factors maintain a balance with the production of oxygen radicals to prevent tissue damage.^18^ However, during times of stress, a large amount of oxygen free radicals are generated, which can disrupt cell metabolism and potentially lead to metabolic diseases. Immunoglobulin, a protein with antibody activity, plays a crucial role in humoral immunity by providing anti-toxicity and antibacterial effects. Studies have shown that the Qizhu Tang decoction, a traditional Chinese herbal formula,composed of four herbal ingredients (Cangzhu, Poria, Panax notoginseng and Astragalus) has been shown to have strong antioxidant activity and is effective in preventing brain oxidative damage in rats.^19^ Wang et al.^20^ found that supplementation with Chinese herbal medicine mixture in feed can increase the activity of postrumen digestive enzymes and enhance serum antioxidant status. The addition of CHM-A and CHM-B to the diet may benefit ruminant production.

In order to enhance the reproductive performance of dairy goats, an experiment was conducted to screen kidney and aphrodisiac herbs such as epimedium, deer antler cream, Eucommia Bark, and others based on the visceral manifestation theory of Chinese veterinary medicine. The experiment also included the administration of yin tonic herbs like angelica, white peony, and Prepared Rehmannia Root, along with supplementary Chinese medicine pieces such as yam, fried white art, magnolia, and gardenia, which nourish the spleen and stomach, clear heat, and regulate the functional activity of the organs and meridians. This experiment focused on dairy goats preparing for parturition to investigate whether Chinese herbal medicine can safely and effectively improve reproductive performance by regulating reproductive hormone levels, antioxidant capacity, and immunity. Additionally, this study provided experimental data to support the application of Chinese herbal medicine in the breeding of dairy goats.

## Materials and methods

### Preparation of YYSBFF

YYSBFF composition: Epimedium 15 g, Cervi Cornu Degelatinatum 15 g, Rehmanniae Radix Praeparata15 g, Pyrolae herba 15 g, Drynaria Rhizome 15 g, Angelicae sinensis radix 10 g, Eucommia Bark 10 g, Spatholobi Caulis 8 g, Atractylodis Macrocephalae Rhizoma, 15 g, Paeoniae radix alba 30 g, Cinnamomi Ramulus 12 g, Mangnolia officinalis Cortex 10 g, Gardeniae Fructus 6 g, yam 20 g, Ostreae Concha 50 g, all purchased from Kunming Luosiwan Chinese herbal medicine wholesale market, Yunnan Province. 120 mesh powder through a sieve, sealed and stored for later use.

### Experimental protocol

In this study, 36 Alpine ewes with postpartum lactation and no reproductive diseases, who tested negative for Brucella, were selected from a dairy goat farm in Yunnan Province. They were randomly divided into two groups: the experimental group (EG) and the control group (CG), with 18 ewes in each group. The trial period lasted for 200 d. The methodological procedures of the study are in line with international guidelines for the ethical use of animals. Supervised by the Life Science Ethics Committee of Yunnan Agricultural University (Acceptance No.: 202207050). The experimental group was fed 2% YYSBFF in two stages. The first stage involved feeding 0.5 g/kg body weight per sheep for 14 days, starting one month postpartum. The second stage involved feeding the same amount 14 d before parturition until the ewes gave birth. Throughout the rest of the trial, the control group was uniformly reared and managed, with consistent feeding conditions and amounts. Artificial insemination was used for breeding. The composition and nutritional level of the basal diet are presented in Table 1.

**Table 1.** Basic diet composition and nutritional level.

Raw material	Content/%	Nutritional level2	Calculate the value
Corn	55.00	Dry matter/%	89.00
wheat bran	10.00	Crude protein/%	17.50
Soybeans, peanut straw, chaff mixed with bran	7.00	Fiber/%	7.00
Concentrate1	28.00	Coarse ash/%	10.00
		Ca/%	1.07
		P/%	0.70
		NaCl/%	4.00

Exegesis: (1) The composition of concentrated feed raw materials includes soybean meal, cottonseed meal, wheat and its by-products, corn by-products, vitamins, mineral elements and their complex (chelate) complexes, amino acids, enzyme preparations, antioxidants, and more. (2) The nutrient level is determined through calculations.

### Assessment of reproductive capacity

During the trial period, various parameters were recorded including the number of ewe estrus, the number of embryos, the number of abortions, the number of goat kids, the birth weight of goat kids, the number of weak goat kids, and the birth type of goat kids (the number of single goat kids, twin goat kids, triple goat kids, and male goat kids). To evaluate the performance, it is necessary to calculate the following rates: ewe estrus rate, embryo acceptance rate, abortion rate, parturition rate, weak goat kid rate, single kid rate, twin kid rate, triple kid rate, and male goat kid rate.^21–23^

Estrus identification: Estrus in ewes can be identified by various signs including excitement, chirping, constant tail wagging, frequent urination, crawling across other ewes, loss of appetite, hyperemia and swelling of the vulva, flushed appearance, vaginal looseness and mucus, and sometimes outflow from the vulva.^24^

$$\text{Estrus rate }=\;\left(\begin{array}{l} \text{number of ewes in heat/}\\ \text{number of test ewes} \end{array}\right)\;\times \;100\%;$$

$$\begin{array}{l} \text{Embryo rate =}\\ \left(\begin{array}{l} \text{number of successful embryo ewes/}\\ \text{number of ewes in heat} \end{array}\right)\;\times \;100\%; \end{array}$$

$$\text{Abortion rate }=\;\left(\begin{array}{l} \text{number of aborted ewes/}\\ \text{number of pregnant ewes} \end{array}\right)\;\times \text{ 100\%;}$$

$$\begin{array}{l} \text{Kidding rate }=\\ \left(\begin{array}{l} \text{number of newborn goat kids/}\\ \text{number of kidding ewes} \end{array}\right)\;\times \;100\%; \end{array}$$

$$\begin{array}{l} \text{Weak goat kid rate }=\\ \left(\begin{array}{l} \text{number of weak goat kids/}\\ \text{number of newborn goat kids} \end{array}\right)\;\times \;100\%; \end{array}$$

$$\begin{array}{l} \text{Single kid rate}=\\ \left(\begin{array}{l} \text{number of ewes that gave birth to singleton/}\\ \text{number of kidding ewes} \end{array}\right)\;\times \;100\%; \end{array}$$

$$\begin{array}{l} \text{Twin kid rate}=\\ \left(\begin{array}{l} \text{number of ewes that gave birth to twins/}\\ \text{number of kidding ewes} \end{array}\right)\times 100\%; \end{array}$$

$$\begin{array}{l} \text{Triple kid rate}=\\ \left(\begin{array}{l} \text{number of ewes that gave birth to triplets/}\\ \text{number of kidding ewes} \end{array}\right)\;\times \;100\%; \end{array}$$

$$\text{Male goat kid rate}=\left(\begin{array}{l} \text{number of male goat kids/}\\ \text{total number of goat kids} \end{array}\right)\;\times \;100\%.$$

The weak goat kid is characterized by an undersized body, thin and frail legs, and difficulty standing or inability to stand after birth.

### Determination of reproductive hormones, antioxidant factors and immunoglobulin levels

In the early morning following the completion of the initial stage of medication, both the experimental group and the control group collected 5 mL of blood simultaneously from the jugular vein. The collected blood was then centrifuged at a speed of 4000 r/min for 8 min to separate the serum, which was subsequently stored at a temperature of −20 °C for future use.

The levels of sex hormones,^25^ such as Follicle Stimulating Hormone (FSH), Luteinizing Hormone (LH), Estradiol (E2), and Progesterone (P4), were determined using a commercial ELISA kit from Shanghai Youxuan Biotechnology Co., Ltd.^26^ Additionally, the levels of antioxidant markers including glutathione peroxidase (GSH-Px), Total Superoxide Dismutase (T-SOD), Catalase (CAT), Malondialdehyde (MDA), as well as immunoglobulin M (IgM), immunoglobulin G (IgG), and immunoglobulin A (IgA) were also determined.^27^^,^^28^

### Monitoring of liver and kidney function

The levels of alanine aminotransferase (ALT), aspartate aminotransferase (AST), total protein (TP), albumin (ALB), urea (UREA, UREA), and creatinine (CREA) were determined in serum using a veterinary automatic blood biochemistry analyzer (Mindray International Co., Ltd.).^29^

### Data statistics and analysis

Excel 2019 was used for preliminary statistical collation of data, followed by statistical analysis using GraphPad Primim8.0.2. The results are presented as mean ± standard deviation. A significant difference is denoted by *p* < .05 (*), while an extremely significant difference is denoted by *p* < .01 (**).

## Results

### YYSBFF improves dairy goat fertility

**Figure 1.**
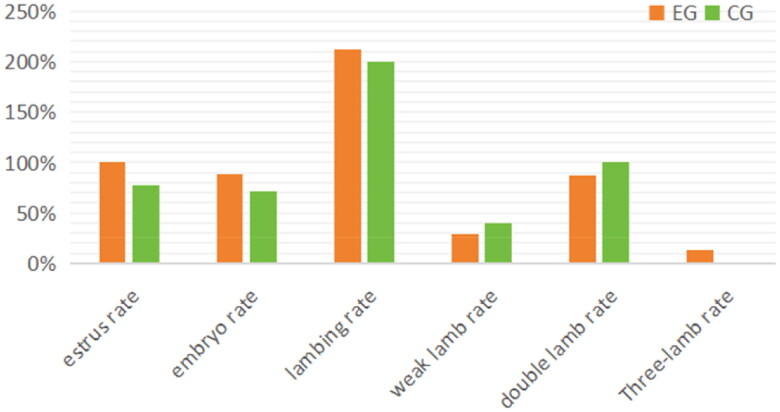
Analysis of reproductive performance indicators.

Estrus rate, embryo rate, kidding rate, and lamb birth weight are all important indicators of ewes’ reproductive performance. The statistical analysis results are presented in Table 2 and Fig. 1. Compared to the control group, the experimental group showed a 22% increase in estrus rate, a 17% increase in embryo rate, a 12.5% increase in kidding rate, and an approximately decrease 10% in the weak goat kid rate. Furthermore, the average birth weight of goat kids increased by around 0.2 kg. No abortions occurred in either group during the entire gestation period, and twins were the predominant birth type. These findings demonstrate a significant improvement in the overall reproductive performance of ewes in the experimental group, with the number of offspring reaching 1.7 times that of the control group. Additionally, the goat kids exhibited greater robustness.

**Table 2.** Statistics of reproductive performance indicators.

Group	Number of estrus	Number of embryos	Number of miscarriages	Number of lambs	Weak lamb number	Double lamb number	Number of three lambs	Average lamb weight/(kg)
EG	18	16	0	34	10	14	2	2.87 ± 0.08
CG	14	10	0	20	10	10	0	2.63 ± 0.05

The graph illustrates the percentage values for various parameters such as estrus rate, embryo rate, kidding rate, weak goat kid rate, twin kid rate, and triple kid rate. The experimental group is represented by the orange bar, while the control group is represented by the green bar.

### Changes in serum reproductive hormone levels

**Figure 2.**
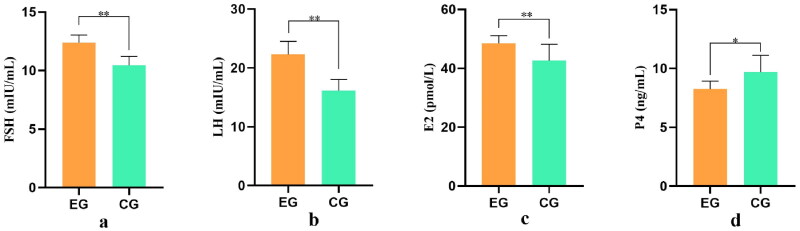
Analysis of differences in serum reproductive hormone levels in ewes.

The role of serum reproductive hormone levels in regulating animal reproduction, development, and growth is crucial. The double-antibody sandwich ELISA was employed to measure the levels of reproductive hormones including follicle-stimulating hormone (FSH), luteinizing hormone (LH), estradiol (E2), and progesterone (P4) in the ewes’ serum. The results indicated that the experimental group had significantly higher levels of FSH and LH compared to the control group. Additionally, the level of E2 was significantly higher in the experimental group (*p* < .05), while the level of P4 was significantly lower (*p* < .05). The increase in FSH and LH promotes follicle growth and development, stimulates the rapid maturation and ovulation of follicles, and, in conjunction with E2, enhances estrus and ovulation in ewes. These findings demonstrate that YYSBFF has the potential to promote estrus and ovulation in ewes. The specific data are shown in Fig. 2.

The ‘a,’ ‘b,’ represent the levels of FSH and LH, respectively, measured in mIU/mL. Similarly, ‘c’ represents the level of E2 measured in pmol/L, and ‘d’ represents the level of MDA, measured in ng/mL. The x-axis represents the groups, and the y-axis represents the level of specific content.

### Serum antioxidant index test results

**Figure 3.**
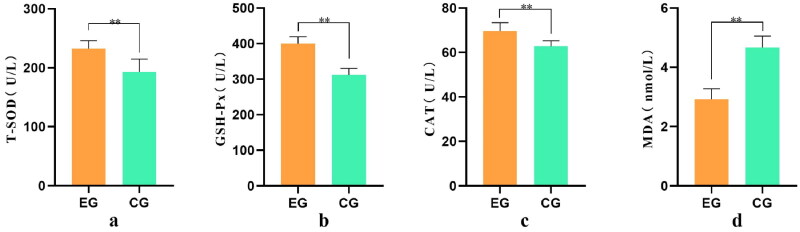
Analysis of antioxidant factor differences.

Superoxide dismutase, glutathione peroxidase, and catalase are the primary enzymes in the animal body’s defense against free radical attack. The experimental results are presented in Fig. 3. It is evident that the T-SOD content in the experimental group was significantly higher than that in the control group (*p* < .05). Moreover, the MDA content was significantly lower than that in the control group (*p* < .01). These findings suggest that the addition of YYSBFF to the feed effectively enhances the antioxidant capacity of dairy goats and reduces stress-induced damage.

The ‘a,’ ‘b,’ and ‘c’ represent the levels of T-SOD, GSH-Px, and CAT, respectively, measured in U/L. Similarly, ‘d’ represents the level of MDA, measured in nmol/L. The x-axis represents the groups, and the y-axis represents the level of specific content.

### Serum immunoindex test results

**Figure 4.**
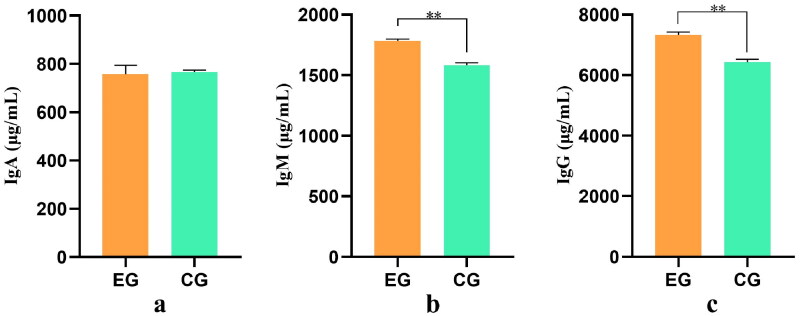
Analysis of serum immunoglobulin level differences.

Immunoglobulins play a crucial role in the body’s defense system and serve as an important indicator of the body’s immune status. The statistical analysis results presented in Fig. 4 focus on measuring the levels of IgM, IgG, and IgA in ewe serum. In the experimental group, the IgM level was significantly higher than that in the control group (*p* < .05). These results confirm that YYSBFF has the ability to enhance the humoral immune function.

The figures ‘a,’ ‘b,’ and ‘c’ represent the content of serum IgA, IgM, and IgG, respectively. The x-axis represents grouped information, and the y-axis represents the level of specific content measured in μg/mL.

### Serum biochemical index test results

**Figure 5.**
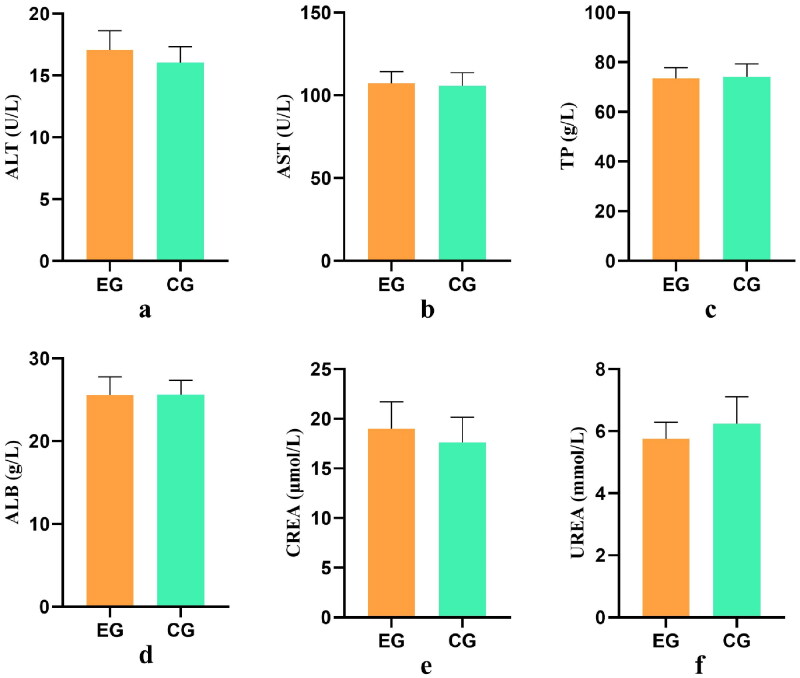
Analysis of differences in serum biochemical indicators.

Blood biochemistry serves as a valuable tool for evaluating changes in animal metabolism and organ function, particularly for assessing abnormal liver and kidney function. The results presented in Fig. 5, indicate that there were no statistically significant differences (*p* > .05) in the levels of ALT, AST, TP, ALB, CREA, and UREA between the experimental group and the control group. This suggests that the addition of YYSBFF to the diet did not have any detrimental effects on the organ functions of ewes and supports the high safety of its clinical application.

The biochemical indexes of ALT, AST, TP, ALB, CREA, UREA are represented by figures ‘a’ to ‘f’, respectively. The x-axis represents the group, and the y-axis represents the level of specific content.

## Discussion

The YYSBFF formula consists of various ingredients including Epimedium, Cervi Cornu Degelatinatum, Rehmanniae Radix Praeparata, Pyrolae herba, Drynaria Rhizome, Angelicae sinensis radix, Eucommia Bark, Spatholobi Caulis, Atractylodis Macrocephalae Rhizoma, Paeoniae radix alba, Cinnamomi Ramulus, Mangnolia officinalis Cortex, Gardeniae Fructus, yam, and Ostreae Concha. According to the fundamental theories of Traditional Chinese Medicine (TCM), Thereinto, Epimedium, Cervi Cornu Degelatinatum, Pyrolae herba, Drynaria Rhizome, and Eucommia Bark, forming the jun medicine of the Compound formulation, have the ability to tonify kidney yang, strengthen muscles and bones, and dispel rheumatism. Rehmanniae radix praeparata, Angelicae sinensis radix, Paeoniae radix alba, Spatholobi Caulis, Atractylodis Macrocephalae Rhizom, and yam has the effect of nourishing the liver and kidney, activating blood circulation, tonifying the spleen and stomach, and balancing water levels. Mangnolia officinalis Cortex, Gardeniae Fructus, and Ostreae Concha have the ability to dissolve dampness, clear internal heat, and reinforce the hidden spirit, acting as an adjuvant. Cinnamomi Ramulus, on the other hand, promotes sweating, dissolves muscles, warms the meridians, and induces menstruation. By promoting the production of qi, an abstract element regarded as enhancing bodily activity in TCM theory, and blood, the YYSBFF formula achieves dynamic regulation and multi-organ effects, enhancing reproductive performance.

The statistical analysis of the reproductive performance indicators of ewes reveals that the increase in the number of goat kids in the experimental group can be attributed to the higher estrus rate and embryo rate. This suggests that the compound Chinese medicine has a positive effect on promoting estrus and ovulation. The significant increase in serum FSH and LH concentrations further supports this conclusion. Although many Chinese herbal medicines do not contain hormones, they have the ability to regulate the body’s hormone levels. For instance, studies have shown that epimedium can increase the levels of LH, FSH, and E2 in animals, as well as upregulate the gene expression of LHR and FSHR in animal ovaries, along with ER genes in uterine tissues.^30^ Icariin, found in epimedium, can stimulate the secretion of FSH and LH by anterior pituitary cells and increase the expression of FSH mRNA and LH mRNA in pituitary cells.^31^ Additionally, a study using a four-object soup (SWT) composed of rehmannia and white peony for mouse gavage demonstrated that it can regulate sex hormone levels through NRF2/HO-1 and STAT3/HIF-1α signaling pathways, thereby promoting follicle development.^32^ In another study, the addition of compound traditional Chinese medicine preparations to the diet of perinatal dairy cows resulted in significantly increased concentrations of FSH and LH in the treatment group, while the concentrations of P4 and E2 were significantly reduced.^33^ A separate experimental study found that YYSBFF significantly increased serum FSH and LH levels. The active ingredients in YYSBFF have been found to exert a hormone-like effect by interacting with the hypothalamic-pituitary-gonadal axis. These ingredients bind to estrogen receptors in dairy goats, resulting in estrogenic effects. They also alter the levels of endogenous FSH, LH, E2, P4, and other sex hormones, which in turn promote the growth and development of follicles, stimulate follicle maturation and ovulation, and ultimately increase estrus rate, embryo rate, and kidding rate.

Modern pharmacological studies have shown that traditional Chinese medicine is closely related to the activity of antioxidant enzymes. The active ingredients in traditional Chinese medicine, such as polysaccharides, have antioxidant functions and enhance cellular immunity. The antioxidant capacity is associated with its ability to scavenge free radicals, reduce oxidative stress, and induce the expression of antioxidant enzyme genes.^34^ Additionally, components like total flavonoids (TFE), alkaloids, and others can activate immune organs, macrophages, B lymphocytes, T lymphocytes, and natural killer (NK) cells. They also promote the secretion of immune factors, complement molecules, and antibodies.^35^^,^^36^ However, the specific mechanism of action of these components on the immune system remains unclear.^37^^,^^38^ Animals’ antioxidant levels are significantly correlated with reproductive performance, making it an important factor in improving animal reproductive performance. Under normal circumstances, the body maintains a dynamic balance between its antioxidant capacity and the production of oxygen radicals, thus preventing tissue damage.^21^ However, when the body is subjected to oxidative stress, a large number of oxygen free radicals are produced, affecting cell metabolism and increasing the risk of oxidative stress-related diseases.^39^ This experimental study found that YYSBFF can increase the concentrations of GSH-Px, T-SOD, and IgG, while reducing the concentration of MDA. This enhances the body’s ability to scavenge oxygen radicals, boosts immunity, and prevents lipid peroxidative degradation. The results indicate that the compound TCM preparation significantly enhances the antioxidant capacity and disease resistance of dairy goats, reducing postpartum stress damage. Similar research shows that Traditional Chinese herbal medicine complex supplementation improves reproductive performance, serum biochemical parameters, and antioxidant capacity in periparturient dairy cows.^33^

Chinese herbal medicine has numerous advantages and therapeutic effects. However, it is important to use it properly to avoid any potential harm to the body. The liver and kidney, being crucial metabolic organs, play a significant role in maintaining overall health. Liver function is often assessed using indicators such as ALT and AST, whereas renal function is evaluated through serum urea and creatinine levels. In this experiment, no significant difference was observed between the experimental group and the control group (*p* > .05), indicating that the compound Chinese medicine did not impair the liver and kidney functions of dairy goats.

This study confirmed the clinical effectiveness of a compound traditional Chinese medicine preparation called YYSBFF. The confirmation was based on observations of reproductive epigenetic indicators in ewes, as well as the measurement of serum reproductive hormones, antioxidant factors, and immunoglobulin levels. The study also provided preliminary insights into the mechanism by which YYSBFF improves the reproductive ability of ewes. However, further exploration in laboratory settings is required to elucidate specific pathways and targets involved. This direction of research holds promise for subsequent in-depth investigations.

## Conclusions

YYSBFF has been found to enhance the reproductive performance of dairy goats by regulating the levels of sex hormones. Additionally, it has been observed to enhance the antioxidant capacity and humoral immunity level of dairy goats. Importantly, it does not have any adverse effects on the liver and kidney function of breeding ewes.

## Data Availability

We encourage all authors of articles published in MDPI journals to share their research data. In this section, please provide details regarding where data supporting reported results can be found, including links to publicly archived datasets analyzed or generated during the study. Where no new data were created, or where data is unavailable due to privacy or ethical restrictions, a statement is still required. Suggested Data Availability Statements are available in section ‘MDPI Research Data Policies’ at https://www.mdpi.com/ethics.

## References

[1] Miller BA, Lu CD. Current status of global dairy goat production: an overview. Asian-Australas J Anim Sci. 2019;32(8 Suppl):1219–1232. 31357263 10.5713/ajas.19.0253 PMC6668863

[2] Pirisi A, Lauret A, Dubeuf J. Basic and incentive payments for goat and sheep milk in relation to quality. *Small Ruminant Res.* 2007;68(1-2):167–178.

[3] Luo J, Wang W, Sun S. Research advances in reproduction for dairy goats. *Asian-Australas J Anim Sci*. 2019;32(8):1284–1295. 31357269 10.5713/ajas.19.0486 PMC6668861

[4] Matsumoto S, Tanaka T, Endo N. Intravaginal administration of estradiol benzoate capsule for estrus. *J Reprod Develop*. 2021;67(2):83–88. 10.1262/jrd.2020-126 PMC8075725 33518696

[5] Khan S, Jamal MA, Khan IM, et al. Factors affecting superovulation induction in goats (*Capra hericus*): An analysis. *Front Vet Sci*. 2023;10:1152103. 37035816 10.3389/fvets.2023.1152103 PMC10079885

[6] Palacios C, Abecia JA, Plaza J, Hidalgo C, de la Fuente LF. Efficiency of artificial insemination at natural ­estrus in organic Churra Ewes. *Vet Sci*. 2022;9(7):370. 10.3390/vetsci9070370 PMC9319334 35878387

[7] Kalds P, Zhou S, Cai B, et al. Sheep and goat genome engineering: from random transgenesis to the CRISPR era. *Front Genet*. 2019;10:750. 31552084 10.3389/fgene.2019.00750 PMC6735269

[8] Pendleton RJ, Youngs CR, Rorie RW, et al. Follicle stimulating hormone versus pregnant mare serum gonadotropin for superovulation of dairy goats. *Small Ruminant Research.* 1992;8(3):217–224.

[9] Baril G, Remy B, Vallet J, Beckers J-F. Effect of repeated use of progestagen-PMSG treatment for estrus control in dairy goats out of breeding season. *Reprod Domest Anim*. 1992;27(3):161–168.

[10] Zheng J, Liang S, Zhang Y, et al. Effects of compound Chinese herbal medicine additive on growth performance and gut microbiota diversity of Zi goose. *Animals*. 2022;12(21):2942. 36359068 10.3390/ani12212942 PMC9655946

[11] Yasmin AR, Chia SL, Looi QH, Omar AR, Noordin MM, Ideris A. Herbal extracts as antiviral agents. *Feed Additives*. 2020:115–132. doi: 10.1016/B978-0-12-814700-9.00007-8.

[12] Zhou X, Seto SW, Chang D, et al. Synergistic effects of Chinese herbal medicine: a comprehensive review of methodology and current research. *Front Pharmacol*. 2016;7:201. 27462269 10.3389/fphar.2016.00201 PMC4940614

[13] Yuan H, Ma Q, Cui H, et al. How can synergism of traditional medicines benefit from network pharmacology? *Molecules*. 2017;22(7):1135. 28686181 10.3390/molecules22071135 PMC6152294

[14] Garverick HA, Smith MF. Female reproductive physiology and endocrinology of cattle. *Vet Clin North Am Food Anim Pract*. 1993;9(2):223–247. 8348369 10.1016/s0749-0720(15)30643-5

[15] Vlčková R, Sopková D, Andrejčáková Z, et al. Dietary supplementation of yucca (*Yucca schidigera*) affects ovine ovarian functions. *Theriogenology.* 2017;88:158–165. 27746005 10.1016/j.theriogenology.2016.09.026

[16] El-Zaher HM, Eid SY, Shaaban MM, et al. Ovarian activity and antioxidant indices during estrous cycle of Barki ewes under effect of thyme, celery and salinomycin as feed additives. *Zygote*. 2021;29(2):155–160. 33228827 10.1017/S0967199420000611

[17] Xia T, Fu Y, Gao H, et al. Recovery of ovary function impaired by chemotherapy using Chinese herbal medicine in a rat model. *Syst Biol Reprod Med*. 2014;60(5):293–303. 24831605 10.3109/19396368.2014.920057

[18] Rahal A, Kumar A, Singh V, et al. Oxidative stress, prooxidants, and antioxidants: the interplay. *Biomed Res Int*. 2014;2014:761264–761219. (). 24587990 10.1155/2014/761264 PMC3920909

[19] Xuejiang W, Ichikawa H, Konishi T. Antioxidant potential of Qizhu Tang, a Chinese Herbal Medicine, and the effect of cerebral oxidative damage after ischemia reperfusion in rats. *Biol Pharm Bull*. 2001;24(5):558–563. 11379780 10.1248/bpb.24.558

[20] Wang H-f, Yang W-r, Wang Y-x, Yang Z-b, Cui Y-h The study on the effects of Chinese herbal mixtures on growth, activity of post-ruminal digestive enzymes and serum antioxidant status of beef cattle. *Agric Sci China.* 2011;10(3):448–455.

[21] Andreoli SP. Reactive oxygen molecules, oxidant injury and renal disease. *Pediatr Nephrol*. 1991;5(6):733–742. 1662982 10.1007/BF00857888

[22] Notter DR, Mousel MR, Leeds TD, Lewis GS, Taylor JB. Effects of rearing triplet lambs on ewe productivity, lamb survival and performance, and future ewe performance. *J Anim sci*. 2018;96(12):4944–4958. 30202943 10.1093/jas/sky364 PMC6276584

[23] Kenyon PA-O, Corner-Thomas RA-O. Breeding Ewe Lambs: An Australasian perspective. *Animals*. 2022;12(22):3207. 36428434 10.3390/ani12223207 PMC9686899

[24] Mohan K, Kumar N. Comparative evaluation of estrus synchronization protocols on reproductive performance and estrus behavior in Barbados Black Belly sheep. *Vet World*. 2023;16(11):2244. 38152269 10.14202/vetworld.2023.2244-2249 PMC10750750

[25] Burrows H. *Biological actions of sex hormones*. Cambridge: Cambridge University Press; 2013.

[26] Mäkelä R, Leinonen A, Suominen T. Analysis of luteinizing hormone (LH): Validation of a commercial ELISA kit for LH analysis and quantification in doping control samples. *Drug Test Anal*. 2020;12(2):239–246. 31655497 10.1002/dta.2716

[27] Su G, Zhou X, Wang Y, et al. Effects of plant essential oil supplementation on growth performance, immune function and antioxidant activities in weaned pigs. *Lipids Health Dis*. 2018;17(1):139. 29903022 10.1186/s12944-018-0788-3 PMC6003089

[28] Tvarijonaviciute A, Martínez-Subiela S, Caldin M, Tecles F, Ceron JJ. Evaluation of automated assays for immunoglobulin G, M, and A measurements in dog and cat serum. *Vet Clin Pathol*. 2013;42(3):270–280. 23919681 10.1111/vcp.12069

[29] Wolford ST, Schroer RA, Gohs FX, et al. Reference range data base for serum chemistry and hematology values in laboratory animals. *J Toxicol Environ Health*. 1986;18(2):161–188. 3712484 10.1080/15287398609530859

[30] Munir N, Mahmood Z, Yameen M, Mustafa G. Therapeutic response of Epimedium gandiflorum’s different doses to restore the antioxidant potential and reproductive hormones in male albino rats. *Dose Response*. 2020;18(3):1559325820959563. 32973420 10.1177/1559325820959563 PMC7493261

[31] Shindel AW, Xin Z-C, Lin G, et al. Erectogenic and neurotrophic effects of icariin, a purified extract of horny goat weed (*Epimedium* spp.) in vitro and in vivo. *J Sex Med*. 2010;7(4 Pt 1):1518–1528. 20141584 10.1111/j.1743-6109.2009.01699.x PMC3551978

[32] Zhou F, Song Y, Liu X, et al. Si-Wu-Tang facilitates ovarian function through improving ovarian microenvironment and angiogenesis in a mouse model of premature ovarian failure. *J Ethnopharmacol*. 2021;280:114431. 34293457 10.1016/j.jep.2021.114431

[33] Ran M, Cha C, Xu Y, et al. Traditional Chinese herbal medicine complex supplementation improves reproductive performance, serum biochemical parameters, and anti-oxidative capacity in periparturient dairy cows. *Anim Biotechnol*. 2022;33(4):647–656. 32930627 10.1080/10495398.2020.1819823

[34] Wang H, Liu YM, Qi ZM, et al. An overview on natural polysaccharides with antioxidant properties. *Curr Med Chem*. 2013;20(23):2899–2913. 23627941 10.2174/0929867311320230006

[35] Xiang X, Cao N, Chen F, et al. Polysaccharide of atractylodes macrocephala koidz (Pamk) alleviates cyclophosphamide-induced immunosuppression in mice by upregulating Cd28/Ip3r/Plcγ-1/Ap-1/Nfat signal pathway. *Front Pharmacol*. 2020;11:529657. 33363462 10.3389/fphar.2020.529657 PMC7753208

[36] Zhang P, Hu L, Bai R, et al. Structural characterization of a pectic polysaccharide from Codonopsis pilosula and its immunomodulatory activities in vivo and in vitro. *Int J Biol Macromol*. 2017;104(Pt A):1359–1369. 28600205 10.1016/j.ijbiomac.2017.06.023

[37] Bendickova K, Fric J. Roles of IL-2 in bridging adaptive and innate immunity, and as a tool for cellular immunotherapy. *J Leukoc Biol*. 2020;108(1):427–437. 32480431 10.1002/JLB.5MIR0420-055R PMC7384134

[38] Cao L-H, Qiao J-Y, Huang H-Y, et al. PI3K–AKT signaling activation and icariin: The potential effects on the perimenopausal depression-like rat model. *Molecules*. 2019;24(20):3700. 31618892 10.3390/molecules24203700 PMC6832648

[39] Sordillo LM, Aitken SL. Impact of oxidative stress on the health and immune function of dairy cattle. *Vet Immunol Immunopathol*. 2009;128(1-3):104–109. 19027173 10.1016/j.vetimm.2008.10.305

